# From standard-setting to interoperability: emergency medical team data in the 2026 Venezuela earthquake response

**DOI:** 10.1016/j.lana.2026.101667

**Published:** 2026-10-14

**Authors:** Attila J. Hertelendy, Felix Holl, Tatsu Kubo, Alberto Jacir, Daniel Hanfling

**Affiliations:** a Department of Information Systems and Business Analytics, College of Business, Florida International University, Miami, FL, USA; b Disaster Medicine Fellowship, Department of Emergency Medicine, Beth Israel Deaconess Medical Center, Boston, MA, USA; c Africa Institute, Neu-Ulm University of Applied Sciences, Neu-Ulm, Germany; d Leibniz Science Campus Digital Public Health, Bremen, Germany; e Global Health Informatics Working Group, American Medical Informatics Association, Washington, DC, USA; f Department of Public Health and Health Policy, Graduate School of Biomedical and Health Sciences, Hiroshima University, Japan; g Department of Multi-Sectoral Preparedness & Strategic Coordination for Health Security, Center for Health Security, Kyoto University, Japan; h Cano Health, Miami, FL, USA; i Venezuela College of Physicians, Caracas, Venezuela; j Department of Emergency Medicine, George Washington University, Washington, DC, USA

On June 24, 2026, twin earthquakes (M7.2 and M7.5) struck northern Venezuela seconds apart, producing the country's deadliest earthquake in more than a century.^1^ The broader public health toll and context are detailed in Paníz-Mondolfi and coauthors viewpoint.^2^ International emergency medical teams (EMTs) deployed alongside national responders,^3^ and a familiar problem recurred: even a system built to produce standardized reports could not feed them cleanly into the platform coordinating the response.

Over the past decade, the global EMT community has invested heavily in standardization. The WHO EMT Minimum Data Set (MDS), endorsed in 2017 and first activated during Cyclone Idai (Mozambique, 2019), was designed to enable timely, comparable reporting across teams and emergencies, replacing the patchwork of bespoke forms that teams had historically brought with them.^4^ The EMT 2030 Strategy highlights information management, interoperability, and integration of digital systems into national coordination.^5^ Considerable effort has gone into aligning EMT processes and records with the MDS, including mapping national platforms onto it.^6^

In Venezuela, international EMTs were coordinated through the Ministry of Health-led Medical Information and Coordination Cell (CICOM), supported by PAHO/WHO, with operational reporting through a virtual web-based platform.^7^ The platform required manual data entry: it could not incorporate standard MDS CSV exports and used indicators and reporting periods misaligned with the MDS. National authorities additionally required identifiable, patient-level information that the MDS does not collect, necessitating a second, parallel stream of data handling. Consequently, a system purpose-built to generate MDS reports still required manual extraction, transformation, and cleaning to satisfy the platform in use, with teams re-keying data, reconciling indicators, and maintaining duplicate records.

These observations do not reflect shortcomings specific to the Venezuelan context, nor do they diminish responders' adaptability under constrained conditions; the accompanying Viewpoint documents the same structural pattern in a different domain.^2^ Comparable divergences incompatible forms, bespoke coordination templates, ministries requesting data the MDS was never designed to hold have recurred since the standard's inception.^4^ They point to a structural gap: agreed global standards still are not systematically integrated into the tools and workflows of live national operations.

Several reasons for that gap are legitimate. Affected states hold sovereignty over health data generated on their territory and every right to define national reporting requirements; identifiable patient-level information may be needed for continuity of care, medico-legal purposes, or surveillance. The principle of data responsibility managing data safely, ethically, and effectively is embedded in humanitarian guidance and rightly places affected populations first.^8^ The problem is not that countries and platforms have needs beyond the minimum dataset; it is that the system offers no reliable mechanism to reconcile them with the common core, so responders build parallel structures instead.

The costs of those parallel structures are consistent and avoidable. Manual re-entry consumes scarce analytic capacity when it is most constrained; every hand-off between incompatible systems introduces transcription and mapping error; and duplicate records amplify the data-protection risks humanitarian frameworks caution against.^8^ Most important, lost interoperability erodes the comparability the MDS exists to deliver: when every response reshapes the data by hand, the standardized, aggregable picture that should span teams and emergencies never materializes.

Closing the gap requires shifting the field's center from defining standards towards ensuring their operational adoption and technical interoperability. We highlight four priorities. First, coordination platforms should ingest machine-readable MDS exports rather than forcing manual entry that discards structure teams have already produced. Second, additional national requirements should be met by mapping and extending the common dataset, not replacing it: Japan's J-SPEED aligns with the MDS while adding national items,^9^ and Indonesia's profiling of the MDS onto its national platform using Fast Healthcare Interoperability Resources shows how country-specific variables can be added as extensions to a shared core rather than a separate system.^6^ Third, interoperability needs governance: data-sharing arrangements and mapping specifications negotiated before a crisis, honoring data sovereignty and continuity-of-care needs while preserving a comparable minimum. Finally, artificial intelligence can bridge residual gaps without adding strain to front-line personnel: optical character recognition digitizes physical records, while large language models parse and translate unstructured data into standardized formats in real time.

The MDS represents a decade of painstaking consensus-building, and its adoption from Cyclone Idai onwards is a genuine advance in disaster health information.^4^ But a standard that must be manually dismantled and rebuilt to fit each platform it encounters is not yet doing the work it was designed for. The next phase of the EMT initiative is less about adding new standards than about making the ones we have survive contact with the field.^10^ The Venezuela response is a reminder that interoperability is not a technical afterthought but a core determinant of whether standardized information and the better-coordinated care it enables reaches people in the rubble.

## Contributors

AJH, led the project, provided oversight, reviewed the literature, wrote the first draft, and coordinated edits. FH, TK, AJ, and DH contributed content, and edits.

## Declaration of interests

DH declares Leadership or fiduciary role in other board, society, committee or advocacy group, paid or unpaid, at National Academy of Sciences (U.S.), Forum on Medical and Public Health Preparedness, Workshop on Data and Situational Awareness.

All other authors declare no competing interests.
